# Novel protein acylations in Alzheimer’s disease: Molecular, mechanisms, biological significance, and diagnostic and therapeutic potentials

**DOI:** 10.1016/j.jare.2025.11.050

**Published:** 2025-11-25

**Authors:** Yue Yang, Zhihua Yu, Rui Gao, Yuehua Zhang, Ziyi Zhang, Xin Zhong, Linchi Jiao, Ke Du, Yuqiang Wu, Minjie Wei, Mingyan Liu

**Affiliations:** a Department of Pharmacology, School of Pharmacy, China Medical University, Shenyang 110122, China; b Drug and Food Inspection and Testing Center, School of Pharmacy, China Medical University, Shenyang 110122, China; c Department of Neurology, The Fourth Affiliated Hospital of China Medical University, Shenyang 110032, China; d Liaoning Medical Diagnosis and Treatment Center, Shenyang 110167, China

**Keywords:** Novel protein acylations, Alzheimer’s disease, Protein post-translational modification, Epigenetic regulation, Metabolism

## Abstract

**Background:**

Alzheimer’s disease (AD) is a prevalent neurodegenerative disorder characterized by complex pathogenesis including amyloid-β (Aβ), hyperphosphorylated tau mediated neurofibrillary tangles (NFTs), energy metabolism disorders, and neuroinflammation, imposing significant burdens on patients’ families and society. Increasing evidence implicates epigenetic modifications, particularly novel protein acylations, encompassing endogenous energy metabolites- mediated lysine succinylation (Ksucc), propionylation (Kpr), malonylation (Kmal), crotonylation (Kcr), butyrylation (Kbu), 2-hydroxyisobutyrylation (Khib), β-hydroxybutyrylation (Kbhb), and glutarylation (Kglu), lactylation (Kla), alongside benzoylation (Kbz) mediated by sodium benzoate metabolites and isonicotinylation (Kinic) induced by isoniazid, playing a pivotal role in AD pathogenesis.

**Aim of review:**

To enhance the mechanistic understanding of novel acylations, this review systematically summarizes the molecular biological significance of novel protein acylations and their involvement in AD pathogenesis and improvement.

**Key scientific concepts of review:**

Novel acylations exert profound effects on chromatin architecture, DNA accessibility, and transcriptional regulation. Moreover, they critically coordinate protein properties and functions including modulating protein degradation, protein stability, enzyme activity, protein-protein interaction, and subcellular localization. Dysregulation of specific novel acylations orchestrates key cellular processes such as neuroinflammation, metabolic dysfunction, and programmed cell death, thereby contributing to AD progression. This review systematically delineates the mechanistic foundations of novel acylation modifications and underscores their molecular significance. Furthermore, we comprehensively synthesize current knowledge on the involvement of these acylations in AD, offering novel perspectives for developing targeted preventive and therapeutic strategies.

## Graphical abstract

**Figure:**
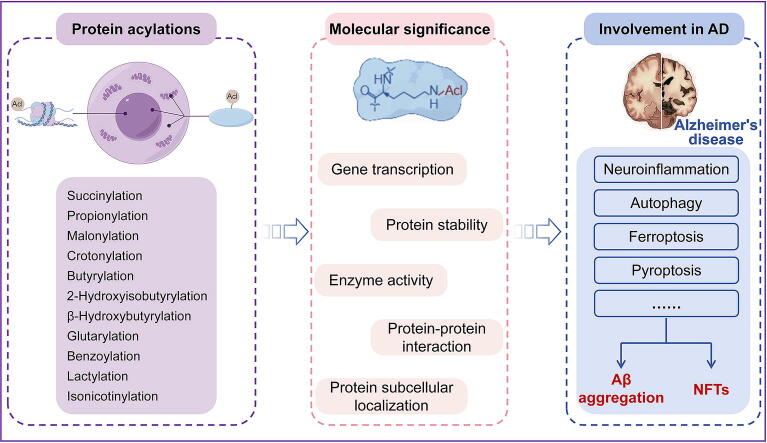

## Introduction

Alzheimer’s disease (AD) is the most prevalent neurodegenerative disorder and constitutes the primary cause of cognitive impairment among the elderly population. With the ongoing demographic shift toward an aging society, the socioeconomic burden of AD is escalating at an unprecedented rate [1]. Clinically, AD manifests as progressive deterioration of cognitive function, neuropsychiatric symptoms, and a loss of independence in performing activities of daily living. The neuropathological hallmarks involve extracellular amyloid-β (Aβ) plaques and intraneuronal neurofibrillary tangles (NFTs) composed of hyper-phosphorylated tau (p-tau), which continue to serve as the gold-standard biomarkers for the disease [2]. Recently, emerging evidence implicates metabolic dysregulation, neuroinflammation, and epigenetic dysfunctions as critical drivers of AD pathogenesis [3,4].

Protein acylation, a critical protein post-translational modification (PTM), is integral to numerous physiological and pathological cellular processes, particularly those associated with neurodegenerative diseases, cancer, and metabolic disorders [5,6]. A significant number of acylations, notably novel lysine acylations such as protein lysine succinylation, propionylation, malonylation, crotonylation, butyrylation, 2-hydroxyisobutyrylation, β-hydroxybutyrylation, glutarylation, benzoylation, lactylation, and isonicotinylation, establish a direct connection between cellular metabolism and both epigenetic remodeling and protein functionality [7]. Novel acylations influence transcriptional programs by modifying chromatin architecture and DNA accessibility. Moreover, they affect protein stability, enzymic activity, protein–protein interactions, and subcellular localization through alterations in protein conformation [6,[8], [9], [10], [11]]. Consequently, these acylations orchestrate energy metabolism, neuroinflammatory signaling, autophagy, and programmed cell death, all of which are critical contributors to AD pathology.

In this review, we offer an extensive analysis of novel acylations, demonstrating the enzymatic processes and regulatory mechanisms. We further delineate the molecular significances of these acylations in the regulation of gene transcription and protein functionality. Moreover, by scrutinizing the regulatory mechanisms involved, this review explores the expanding role of novel protein acylations in AD, assesses their potential as biomarkers for early diagnosis and prognosis, and highlights emerging therapeutic strategies targeting disrupted acylation networks. We also propose future research directions to translate these insights into clinical applications, aiming to enhance our understanding of AD pathogenesis and foster the development of targeted interventions.

## Discovery and principles of novel protein acylations

In recent years, the landscape of PTMs has expanded significantly with the identification of diverse non-acetyl protein acylations. These novel protein acylation modifications constitute a dynamic layer of cellular regulation driven by metabolic intermediates. They involve the covalent attachment of acyl groups to lysine residues through enzymatic or non-enzymatic mechanisms, significantly influencing transcriptional regulation, protein structure and functions. This section systematically explores the discovery, molecular principles, and enzymatic machinery governing these novel acylations, highlighting their evolutionary conservation and broad biological implications.

### Succinylation

**Fig. 1:**
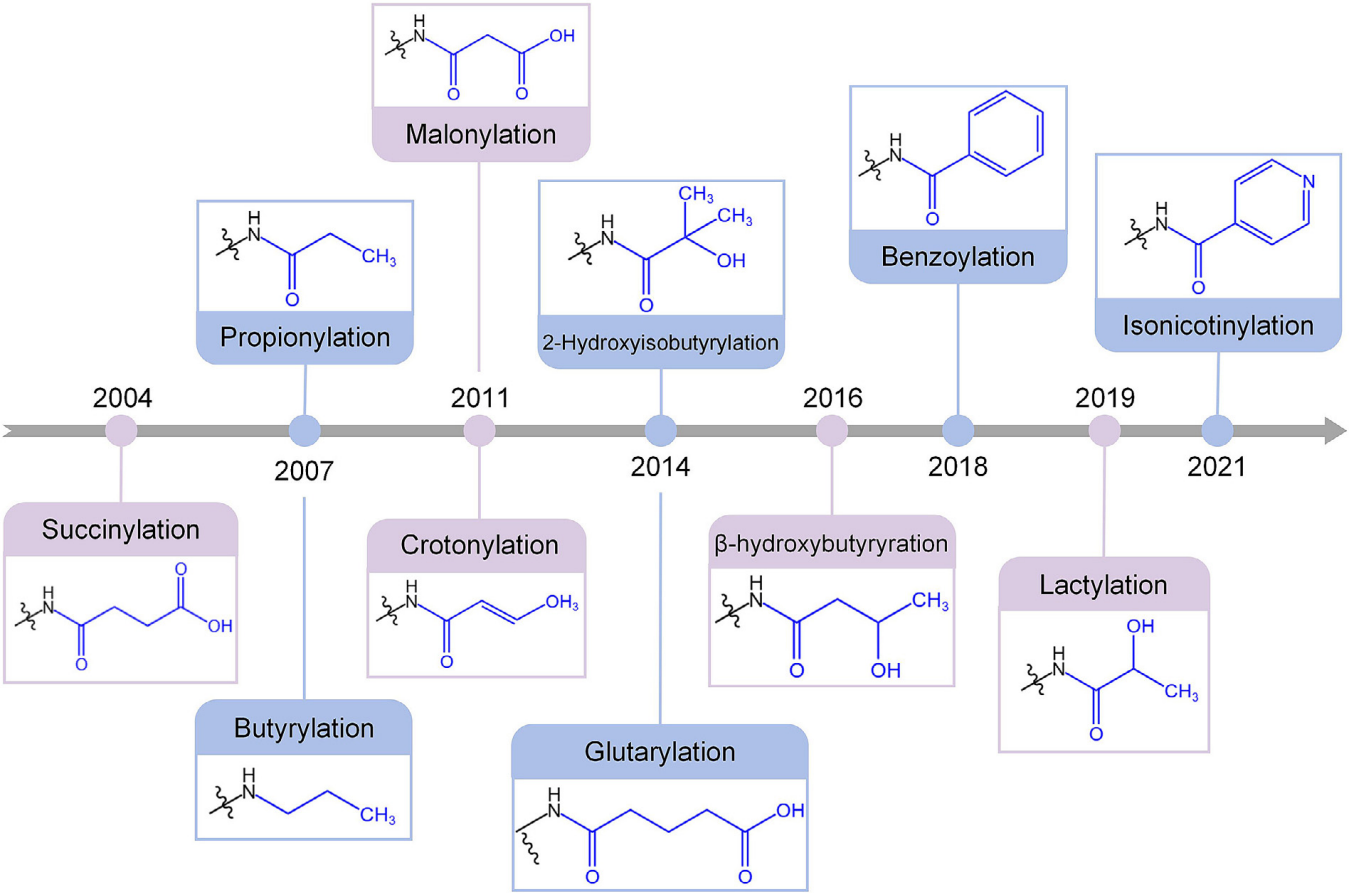
The historical milestones and schematic illustration of novel protein acylations. Novel protein acylations, represented by succinylation, has been identified since 2004, and subsequently, propionylation, malonylation, crotonylation, butyrylation, 2-hydroxyisobutyrylation, β-hydroxybutyrylation, glutarylation, lactylation, alongside benzoylation, and isonicotinylation, have been successively identified.

**Fig. 2:**
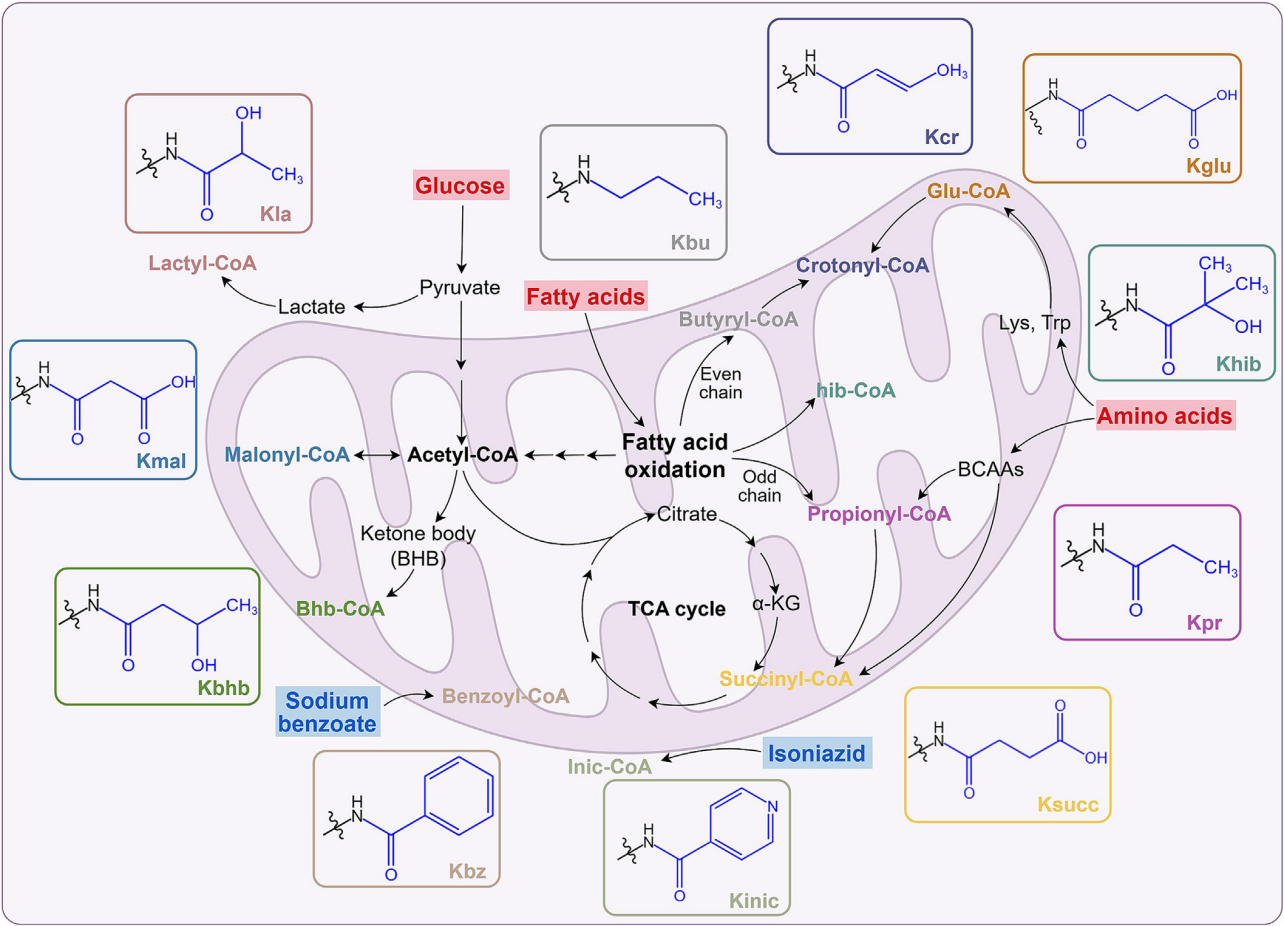
The sources of novel actyl groups based on different metabolism and corresponding novel protein acylations. The metabolism of glucose, fatty acids and amino acids, as well as drugs sodium benzoate and isoniazid, produces corresponding acyl-CoA, providing acyl donors for novel protein acylations. (Abbreviation: CoA: coenzyme A; TCA: tricarboxylic acid; α-KG: α-Ketoglutaric acid). This figure is generated with FigDraw (https://www.figdraw.com).

Succinylation, a novel protein acylation first identified in *Escherichia coli* in 2004, widely presents in various cells and exhibits a high evolutionary conservation [12]. Protein succinylation is currently found to mainly occur on lysine, named lysine succinylation (Ksucc), describing the process of the covalent attachment of the succinyl group derived from succinyl-CoA to the lysine residue (Fig. 1 and Fig. 2). It occurs in the nucleus, cytoplasm, and mitochondria, playing a significant role in regulating gene expression as well as shaping protein structure and function [12,13].

Ksu includes two types: non-enzymatic and enzymatic processes. Initially, Ksu was considered as a non-enzymatic process driven by succinyl-CoA, which is an essential metabolic intermediate predominantly generated in tricarboxylic acid (TCA) cycle in mitochondria [14]. Non-enzymatic Ksu exhibits tissue specificity due to its dependence on the concentration of succinyl-CoA [15]. In addition to non-enzymatic Ksucc, this modification is also positively regulated by some enzymes within the succinyl-CoA metabolic pathway, also recognized as lysine succinyltransferases (LSTase) or writers of succinylation afterwards (Table 1). For example, α-ketoglutarate dehydrogenase complex (α-KGDHC), as a key enzyme in TCA cycle, not only mediates the generation of succinyl-CoA but also directly enzymatic succinylation through its role as a succinyltransferase [13]. Moreover, lysine acetyltransferase 2 A (KAT2A), which belongs to glycine-rich nucleic acid-binding proteins and acetyltransferases (GNAT) family, combines with α-KGDHC, forming complex to obtain succinyl-CoA and subsequently facilitate succinylation [13]. Unlike α-KGDHC and KAT2A, carnitine palmitoyltransferase 1 A (CPT1A), a mitochondrial enzyme involved in acylcarnitine synthesis, acts as lysine succinyltransferase and uses succinyl-CoA as a substrate to succinylate proteins without influencing succinyl-CoA levels [16]. Furthermore, p300/CREB-binding protein (p300/CBP) can induce succinylation on synthetic histone tail peptides in vitro, also serving as a potential succinyltransferase [17]. The currently known desuccinylases mainly include Sirt5 and Sirt7, which have been reported to mediate desuccinylation of histone and non-histone proteins [18,19]. Accumulating evidence suggests that protein Ksu is involved in gene transcription, immune response, and cellular metabolism. Moreover, the misregulation of succinylation is associated with various diseases, such as cancer and neurological diseases [17,18].

**Table 1:** Regulatory enzymes of novel protein acylations identified in mammals.

Novel acylation type	Writers	Readers	Erasers	Modified substrates and corresponding sites
Succinylation	p300/CBP, HAT1, α-KGDHC, CPT1A, GCN5 (KAT2A)	Bromodomains, YEATS domain	Sirt5/7	Histone (H3K23/79/122), RAB7A, PDHC, PKM2-K311, CTBP1-K46/280, LDHA-K188/222, S100A10-K47, MFF-K302, PGAM1-K99, ECHA-K351, ACOX1, PDHA1-K351, SDHA-K547, ANXA1-K166, PRDX3-K84, CS-K393/395, OPTN-K108, PRMT5-K387, SERCA2a-K352, HSD17B10-K99
Propionylation	p300/CBP, GCN5 (KAT2A), PCAF (KAT2B), KAT6A (MOZ), HBO1 (KAT7), KAT8 (MOF)	Bromodomain (BRD4); YEATS domain	Sirt1 ∼3/5, HDAC	Histone (H3K14/23), Tropomodulin-3-K255, Sod2-K132
Malonylation	GCN5 (KAT2A)	N/A	Sirt3/5	Histone, mTOR-K1218, GAPDH-K213, mDia2-K27, ACC1-K1523, VDAC-K46, NCL-K124/398, AGO2-K440, GSTP1-K121
Crotonylation	p300/CBP, GCN5 (KAT2A), PCAF (KAT2B), KAT6A (MOZ), HBO1 (KAT7), KAT8 (MOF), TIP60	YEATS domain (YEATS2, AF9, TAF14), Bromodomain (BRD9, TAF1), DPF2	HDAC1 ∼3, HDAC6 ∼8, Sirt1 ∼3/6/7	Histone (H3K4/9/14/18/23/27, H4K8/12), MTHFD1-K354/553, Lamin A-K265/270, 14–3–3ε-K73/78, PHF5A-K25
Butyrylation	p300/CBP, HBO1 (KAT7), KAT8 (MOF)	Bromodomains (BRD4 BRD9, CECR2, TAF1), YEATS domain	Sirt2/6/7, HDAC11	Histone (H3K14/23/36/37, H4K5), HSP90-K754
2-Hydroxyisobutyrylation	p300/CBP, KAT5, HBO1 (KAT7), Tip60	N/A	Sirt3/7, HDAC2/3	Histone (H3K14/79, H4K5/8), ALDH1A1-K260, ENO1, NAT-K823, EBP1-K210, HXK1-K418, DLD
β-Hydroxybutyrylation	p300/CBP,	ENL	Sirt1 ∼3, HDAC1 ∼3	Histone (H3K9/18/27, H4K8), AHCY-K188/389/405, CS-K393/395, SUCLG1-K81, p53-K120/319/370, STAT1-K592/679, ALDOB-K108, SLC25A5-K147/166, PHD2-K239/385
Glutarylation	p300/CBP, GCN5 (KAT2A)	N/A	Sirt5/7	Histone (H4K91), CPS1, GLUD1, NRF2, OADH-K155/818
Benzoylation	p300/CBP, HBO1 (KAT7)	N/A	Sirt2/3	Histone (H2A, H2B, H3, H4), PPIF-K73/197, ACLY-K978
Lactylation	p300/CBP, Tip60, KAT2A, KAT2B, HBO1 (KAT7), KAT8, NAA10, AARS1, AARS2	Brg1, DPF2	Sirt1 ∼3, HDAC1 ∼3	Histone (H3K18, H4K12), APP-K612, Tau-K677
Isonicotinylation	p300/CBP, Tip60	N/A	HDAC3/6/8	Histone (H2A, H2B, H3, H4), SMAD3-K378

N/A indicates not available. CBP: CREB-binding protein; HAT1: histone acetyltransferase 1; α-KGDHC: α-ketoglutarate dehydrogenase complex; CPT1A: carnitine palmitoyltransferase 1A; GCN5: general control non-depressible 5; KAT: lysine acetyltransferase; YEATS: Yaf9, ENL, AF9, Taf14, and Sas5; Sirt: Sirtuin; RAB7A: Ras-related protein Rab-7A; PDHC: pyruvate dehydrogenase complex; PKM2: pyruvate kinase M2; CTBP1: C-terminal binding protein 1; LDHA: lactate dehydrogenase A; MFF: mitochondrial fission factor; PGAM1: phosphoglycerate mutase 1; ECHA: enoyl-CoA hydratase; ACOX1: acyl-CoA oxidase 1; PDHA1: pyruvate dehydrogenase E1 α1; SDHA: succinate dehydrogenase iron-sulfur subunit; ANXA1: annexin A1; PRDX3: peroxiredoxin 3; CS: citrate synthase; OPTN: optineurin; PRMT5: protein arginine methyltransferase 5; SERCA2a: sarcoplasmic/endoplasmic reticulum Ca^2+^ ATPase 2a; HSD17B10: hydroxysteroid 17-β dehydrogenase 10; PCAF: p300/CBP-associated factor; HBO1: HAT binding to ORC1; HDAC: histone deacetylase; Sod2: superoxide dismutase 2; GAPDH: glyceraldehyde-3-phosphate dehydrogenase; mDia2: mammalian diaphanous 2; ACC1: acetyl-CoA carboxylase 1; VDAC: voltage-dependent anion channel; NCL: nucleolin; AGO2: argonaute 2; GSTP1: glutathione S-transferase Pi 1; MTHFD1: methylenetetrahydrofolate dehydrogenase 1; PHF5A: PHD finger protein 5A; ALDH1A1: Aldehyde dehydrogenase 1 family member A1; ENO1: enolase 1; NAT10: N-acetyltransferase 10; EBP1: ErbB3 binding protein 1; HXK1: hexokinase-1; DLD: Dihydrolipoamide dehydrogenase; ENL: Eleven-nineteen leukemia; AHCY: S-adenosyl-L-homocysteine hydrolase; CS: citrate synthase; SUCLG1: succinate-CoA ligase subunit alpha; STAT1: signal transducer and activator of transcription 1; ALDOB: aldolase B; Fructose-bisphosphate; PHD2: prolyl hydroxylase domain Protein 2; CPS1: carbamoyl phosphate synthase 1; OADH: oxoadipate dehydrogenase; PPIF: protein peptidyl-prolyl cis-trans isomerase F; ACLY: ATP-citrate lyas; APP: amyloid precursor protein.

### Propionylation

Protein lysine propionylation (Kpr) was first discovered in 2007 by Chen et al. and occurred on the lysine of histones [20] (Fig. 1). Propionyl-CoA, an intermediate in fatty acid and amino acid metabolism, act as substrates and donates the propionyl moieties for Kpr, coupling cellular metabolism with chromatin structure and function [21] (Fig. 2). This acylation modification is chemically similar to lysine acetylation (Kac), with the addition of extra carbon atoms, indicating that it may exerts similar biological effects as Kac.

Kpr is also a reversible modification catalyzed by enzymes, including propionyltransferase such as p300/CBP, lysine acetyltransferase 2A (KAT2A), KAT6, KAT7, MOF, MOZ, p300/CBP-associated factor (PCAF), and depropionylase such as Sirt1-3 and Sirt5 (Table 1). The first report of p300/CBP accompanying propionylation was identified as catalyzing this modification process [20] Subsequently, researchers found that KAT2A and PCAF can act as acyltransferase mediated propionylation of histone H3 Lys14 (H3K14) [21]. In addition, KAT6A, a member of MYST (MOZ, Ybf2/Sas3, Sas2, and Tip60) histone acetyltransferase, catalyze histone H3 at lysine 23 (H3K23) propionylation in vitro and in vivo in developmental disorders and various cancers by regulating gene transcription [22]. KAT7, also known as HAT binding to ORC1 (HBO1), serves as a reader of histone Kpr to promote gene expression [23]. However, Sirt family members induced histone propionylation, implying their potential of depropionylase activity [24]. Besides, histone deacetylase (HDAC) enzymes may also serve as potential depropionylase, as evidenced by the presence of propionylation induced by histone deacetylase inhibitors [25]. Researchers suggest a possible association of abnormal propionylation with neurodevelopmental disorder, metabolic disorders and oncological diseases [22,25].

### Malonylation

Protein lysine malonylation (Kmal) is a dynamic and evolutionarily conserved post-translational modification (PTM) first discovered by Yingming Zhao's research team in 2011, and has been observed in both mammalian and bacterial cells [26] (Fig. 1). Malonyl-CoA, converted from acetyl-CoA in an ACC1-dependent manner, acts as the malonyl donor to link to lysine (Fig. 2). Subsequently, modification sites of histone Kmal were identified in HeLa and *S. cerevisiae* cells, respectively, increasing the potential combinatorial diversity of histone PTMs and suggesting a possible new link between histone biology and metabolism [27].

The research on enzymes related to Kmal is still limited. Currently, KAT2A has been reported as a malonyltransferase (writer) as well as Sirt3 and Sirt5 as demalonylases (erasers) catalyzing Kmal in mammalian cells [8,26,28,29] (Table 1). KAT2A promotes the transfer of malonyl to histone lysine from malonyl-CoA and mediates histone Kmal in K562 cells [28]. Argonaute2 (AGO2) malonylation could be regulated by a cytoplasmic-localized short isoform of Sirt3 through a previously unknown demalonylase function [29]. While Sirt5 also serves as a potential demalonylase to mediate histone demalonylation and demalonylation of glutathione S-transferase P1 (GSTP1) [8,26,28].

Kmal exerts regulatory effects on protein activity, stability, interactions, and translocation, as well as gene translation, which all determine its close relationship with various biological processes such as energy metabolism, immune inflammatory response, and programmed cell death, as well as involvement in multiple pathological condition including cancer, myocardial injury, and some other metabolic and inflammatory diseases [30]. For instance, Sirt5 loss-induced hypermalonylation of glutathione S-transferase P (GSTP1) diminishes its protein stability, which aggravates high glucose-induced oxidative stress and mitochondrial dysfunction in cardiomyocytes, and intensified cardiomyocyte senescence, pyroptosis, and DNA damage, exacerbating myocardial injury in diabetic cardiomyopathy [8]. GAPDH malonylation on K213 leads to its dissociation from TNFα mRNA and promotes TNFα translation, inducing inflammation in macrophages [31]. It follows that Kmal is widely implicated in multiple cellular biological processes in various physiology and pathology.

### Crotonylation

Crotonylation is an emerging conservative acylation modification first identified to occur on histone lysine in 2011 [32] (Fig. 1). Multitudinous histone Kcr has been detected to be enriched in the transcriptional start sites (TSSs) and enhancer regions to regulate gene expression [32]. Crotonylation is the transfer of a donor crotonyl group to a lysine residue of proteins, and is regulated by various pathways due to the production of crotonyl-CoA is controlled by diverse pathways. Acyl-CoA synthetase short-chain family member 2 (ACSS2), exogenous supplement of crotonate, butyrate and glutaryl-CoA all play an important role in the conversion of crotonyl-CoA, and subsequently impacts the occurrence of histone and non-histone Kcr [33] (Fig. 2).

The Kcr modification dynamic balance is also prominently regulated by enzyme-dependent Kcr writers, readers, and erasers (Table 1). Up to now, several histone acetyltransferases (HATs) have been identified to be related to Kcr and act as Kcr crotonyltransferases (writers). p300/CBP has been reported to regulate lysine crotonylome by quantitative crotonylome analysis, and p300/CBP catalyzed histone Kcr promotes gene transcription more effectively than extensively studied histone Kac [34]. Moreover, the member of MYST family members such as KAT8 (MOF), KAT2B (PCAF), and KAT7 (HBO1) also possesses crotonyltransferase activity for histone and non-histone Kcr and this activity is conserved in evolution [35,36]. In addition to writers, YEATS domain, bromadomain, as well as double PHD finger (DPF) are also key enzymes regulating Kcr. YEATS is the first identified effective reader of histone Kcr in 2016 [37]. Interestingly, the DPF domain of MOZ (also known as KAT6A) could selectively recognize H3K14cr [38]. Eraser is responsible for removing crotonyl group from the modified proteins. HDACs, including class I (HDAC1, 2, 3, and 8), class II (HDAC6, 7), and class III (Sirt1∼7), have been identified to possess histone decrotonylase activity [39,40]. However, these erasers tend to mediate the decarboxylation of different proteins and sites. For instance, class I HDACs and Sirt1 share the same catalytic center histone deacetylation [41].

Until now, crotonylation has been revealed to play an essential role in multiple biological processes such as gene regulation, protein structure and functions, as well as DNA repair, metabolic reprogramming and immune remodeling [42]. Furthermore, the abnormal crotonylation is related to the development of various diseases including neurological and psychiatric diseases such as AD, cardiovascular diseases, metabolic diseases, as well as various cancers [39,43].

### Butyrylation

Butyrylation is a novel acylation discovered with Kpr simultaneously in 2007 by Zhao et al [20] (Fig. 1). It refers to a biochemical interaction wherein the butyryl group covalently modifies the lysine of proteins using butyryl-CoA derived from even-chain β-oxidation as the substrate (Fig. 2). Notably, Kbu is regulated by gut microbiota due to the high concentrations of short chain fatty acids (SCFAs), which are deposited onto the TSSs of chromatin and exert role of gene regulation [44].

Several regulatory enzymes have been identified as playing an essential role in Kbu (Table 1). p300/CBP was identified to catalyze histone H4 to undergo Kbu and served as potential Kbu writer [20]. HBO1, a lysine acetyltransferase, is another butyryltransferase for catalyzing Kbu modification, especially Kbu of histones H3 and H4 [23]. Additionally, KAT8 has been revealed to be the writer of butyrylation of HSP90 at lysine754 (K754bu) in tumor cells and tissues [45]. Furthermore, Kbu is recognized by some readers including bromodomains of BRD4, BRD9, CECR2, and TAF1. BRD4 could bind and recognize Kbu, especially histone H3, functioning as reader of Kbu, while the reading activity is significantly lower than those of Kac [46]. Additionally, bromodomains of BRD9, CECR2, and the second bromodomain of TAF1 also recognize the longer butyryl mark, possessing the potential Kbu-reading properties [47]. Sirt7 is one of the most important debutyrylation enzymes. It could efficiently remove the butyryl groups of H3K36 and H3K37 [48]. Sirt2 and Sirt6 selectively remove butyryl group more than acetyl group, whereas Sirt1 and Sirt3 showed the opposite selectivity [48]. Recent study has demonstrated that HDAC11 acts as eraser of HSP90 K754bu [45].

Kbu has been demonstrated to induce chromatin remodeling, regulate gene expression, change protein properties and functions under multiple physiological and pathological conditions. It is involved in diverse cellular processes including influences metabolic reprogramming [49]. Aberrant Kbu has been gradually uncovered to be linked to the onset and progression of several diseases such as cancers [45].

### 2-Hydroxyisobutyrylation

2‐Hydroxyisobutyrylation was identified as a novel post-translational modification in 2014 by Dai et al. in male germ cells [50] (Fig. 1). It refers the transfer of a 2‐hydroxyisobutyryl group from 2‐hydroxyisobutyryl‐CoA to lysine residues of proteins (Fig. 2). The initial Khib is found to occur on histones, such as the 2‐hydroxyisobutyrylation of histone H4 lysine 8 (H4K8hib), and is associated with active gene transcription and the regulation of chromatin functions. Subsequently, as research on Khib continued to deepen, Khib has been gradually identified to occur on non-histones, which influence protein structure and functions [51,52].

The modification process of Khib also depends on enzyme regulation, including 2‐hydroxyisobutyryltransferases (writers) and de-2‐hydroxyisobutyrylases (erasers) (Table 1). p300 is the earliest reported 2‐hydroxyisobutyryl transferase in Khib, which could transfer 2‐hydroxyisobutyryl groups onto lysine residues of histones and non-histone [52]. Subsequently, Tip60, also known as KAT5, in human has been identified to catalyze Khib of proteins both in vitro and in vivo [53]. Additionally, KAT family members including such as KAT7 has been identified as novel writer for Khib. KAT7 functions as writer for 2‐hydroxyisobutyrylation of N-acetyltransferase 10 (NAT10) at K823 [51]. Moreover, individual Sirt members and HDAC members exhibit de-2‐hydroxyisobutyrylation. For instance, Sirt3 exhibits unprecedented role in controlling early renal development through mediating de-2-hydroxyisobutyrylation [54]. Sirt7 acts as eraser for Khib removal of NAT10. Khib modification could also be regulated by HDAC3, which serve as potential eraser of Khib [55].

Deep proteomics analysis of Khib has identified thousands of Khib in mammalian cells with different subcellular localizations and participated in a wide range of molecular functions and cellular processes. Furthermore, Khib has been reported to be involved in various physiological processes, such as the maturation and development of sperm [50]. While the dysregulated Khib is associated with onset or progression of various cancer, metabolic diseases, cardiovascular diseases such as cardiac hypertrophy and atrial fibrillation, abnormal kidney development, as well as hepatitis-B virus (HBV) infection [54,55].

### β-Hydroxybutyrylation

β-Hydroxybutyrylation was first discovered by Yingming Zhao and co-workers in 2016 and especially occurred on histone [56] (Fig. 1). A growing number of histone Kbhb sites have been detected in histones including linker histone H1, H2A, H2B, H3, and H4 in mammalian cells [56,57]. Kbhb refers to the linking of β-hydroxybutyryl groups derived from β-hydroxybutyric acid (BHB) and β-hydroxybutyrate (β-OHB), the main component of ketone bodies, to lysine residues of proteins in an enzyme-dependent manner [56] (Fig. 2). Histone Kbhb represents a new type of histone mark linking metabolism to gene expression. With the continuous deepening of research on Kbhb, it has been discovered that Kbhb not only occur on histones, but also on non-histones, and are mainly enzymes related to energy metabolism [58]. Notably, non-histone Kbhb mainly contributes to the alteration of protein properties and functions such as protein stability and activity [59,60].

In addition to the key enzymes of ketone body metabolism and metabolic substrates, Kbhb is influenced by writers, readers, and erasers. p300 is the first identified Kbhb writer to catalyze the addition of β-hydroxybutyryl groups to lysine of histone, including H3K9, H3K18, H3K27, H4K8 sites [57]. As for the reader, till now, only one known Kbhb reader, eleven-nineteen leukemia (ENL), has been identified. Eleven-nineteen leukemia (ENL) recognizes H3K9bhb and associates with it at gene promoters to boost transcription [61]. However, several Kbhb erasers have been reported so far. class I HDACs and class III HDACs have been recognized to possess enzymatic activity for de-β-hydroxybutyrylation, maily involving zinc-dependent HDAC1, HDAC2, as well as NAD^+^-dependent Sirt3. Intriguingly, Sirt3 displays class-selective histone de-β-hydroxybutyrylase activities with preference for H3 K4, K9, K18, K23, K27, and H4K16, but not for H4 K5, K8, K12, which distinguishes it from the zinc-dependent HDACs [62]. Besides, the resent study has further found that HDAC1 to HDAC3 and Sirt1 and Sirt2, exhibited notable de-Kbhb activity toward core histones in vitro [57] (Table 1).

Accumulating evidence has unveiled that Kbhb plays an essential role in regulating biological processes such as metabolism and immune cell function [58,60]. The abnormal Kbhb is strongly associated with human pathophysiological states, including cancers, neurological disorders, cardiovascular diseases, and metabolic diseases [58,60]. Further exploration of the role and mechanism of Kbhb in health and disease will contribute to provide novel insights for disease diagnosis and treatment.

### Glutarylation

Glutarylation was first reported by Yingming Zhao and the co-workers in 2014 [63] (Fig. 2). Kglu represents the process of glutaryl group, which is derived from CoA, an important intermediate in the metabolism of lysine and tryptophan, transfer to protein lysine residues. prefers to modifies meta bolic enzymes and mitochondrial proteins identified by the proteome-wide analysis. More and more Kglu proteins and corresponding sites are gradually being revealed with the development of predictive models including Deepro-Glu, ProtTrans-Glutar and RF-GlutarySite [[64], [65], [66]].

At present, there are relatively few reports on Kglu, with limit identified regulatory enzymes. p300 and GCN5 (KAT2) are known as writers of Kglu, while the glutaryl group is primarily be removed by Sirt5 and Sirt7 in an NAD^+^-dependent manner [63] (Table 1). KAT2 has been reported to mediate H4 Lys91 glutarylation (H4K91glu), which is a histone mark in regulating chromatin structure and dynamics in response to DNA damage, while Sirt7 serves as an eraser of H4K91glu [67]. Sirt5 functions as a lysine deglutarylase to target carbamoyl phosphate synthase 1 (CPS1) for deglutarylation [63].

Kglu has been reported to be involved in transcriptional regulation and some other regulatory processes [67,68]. Aberrant Kglu is associated with tumorigenesis and anti-tumor immunity[68]. The functions of Kglu will be further comprehensively revealed through the application of technologies such as genetic code expansion.

### Benzoylation

Benzoylation is stimulated by sodium benzoate (SB), an FDA-approved drug and a widely used chemical food preservative through generating benzoyl CoA. It was first reported by Yingming Zhao’s team in 2018 [69] (Fig. 1). Kbz is also a novel histone mark linking metabolism and epigenetics by modulating chromatin structure and functions. Till now, only p300/CBP and HBO1 (KAT7) are among the few identified benzoyltransferases serving as writer of Kbz in mammalian cells [70] (Table 1). Many of the numerous Kbz sites identified in mammalian cells are substrates targeted by HBO1. In addition, Sirt2 is the earliest identified Kbz eraser possessing the debenzoylase activity to histone Kbz both in vitro and in vivo [69].

Kbz has been proven to be associated with various processes, including chromatin remodeling, transcription regulation, immune regulation, and tumor growth. Nonetheless, the substrate proteins, regulatory enzymes, regulatory mechanisms and biological functions of Kbz remain largely unknown. More detailed and in-depth studies are expected to further investigate Kbz for providing a basis of clarification of disease mechanisms and development of therapeutic strategies.

### Lactylation

Lactylation is a novel post- translational acylation discovered in 2019 by Yingming Zhao and the co-workers [71] (Fig. 1). Kla occurs through covalent linkage between lactyl-CoA derived from lactate and lysine residues (Fig. 2). It is first reported to occur on histones. Subsequently, accumulating evidence has revealed an increasing number of histone and non-histone Kla and their corresponding sites. Notably, Kla includes two forms: canonical enzymatic lactylation involves lactyl-CoA synthetase-mediated conversion of lactate to lactyl-CoA, and the non-enzymatic D-lactylation-driven by glyoxalase II substrate S-D-lactoylglutathione (SLG) derived from non-lactate sources [72,73].

Currently, various Kla writers have been identified and recognized (Table 1). For example, p300/CBP was the earliest identified lactyltransferase along with the first report of histone Kla [71]. Additionally, it has also been found to catalyze the non-histone protein Kla. With the continuous deepening of Kla research, more and more writers have been identified, including GNAT family members like KAT2A (GCN5) and KAT2B, MYST family members like KAT5 (Tip60), KAT7 (HBO1), and KAT8, aminoacyl-tRNA synthetases (AARS) family members like AARS1, AARS2, as well as -α-acetyltransferase 10 (NAA10). These writers prefer to catalyze Kla modifications of different types of proteins, possibly due to the different cellular localization and substrates. For instance, GNAT family members such as GCN5 and KAT2B, as well as MYST family members such as Tip60, HBO1 and KAT8 are mainly distributed in the nucleus and utilize lactyl-CoA as a substrate to exert lactyltransferase activity [[74], [75], [76], [77], [78]]. NAA10 functions as the lactytransferase and mediates NOL1/NOP2/Sun domain family member 2 (NSUN2) lysine 508 lactylation (K508la) by using lactyl-CoA as the lactyl source [79]. While AARS1 and AARS2, mainly located in the cytoplasm and mitochondrial matrix, moonlight as bona fide lactyltransferases that directly use lactate and ATP to catalyze protein lactylation [80]. These evidences fully demonstrate that different types of writers specifically catalyze Kla modification of various proteins.

Currently, Kla has been extensively studied by researchers and revealed to exerted various biological significance, such as gene transcription, protein properties, location and functions regulation [11,71]. Kla plays an indispensable role in many physiological processes such as embryonic and neural development, as well as somatic cell reprogramming. Dysregulated Kla is involved in many pathological processes and diseases including cancer, neurological disorders, cardiovascular diseases, metabolic disorders, and immune inflammatory diseases [71,74,76]. Consequently, intervening in lactate has become a potential treatment strategy for certain diseases.

### Isonicotinylation

Isonicotinylation, also called 4-picolinylation, was first reported by the research group of Professor Hongquan Zhang in 2021 [81] (Fig. 1). The researchers found that isoniazid (INH), a first-line anti-tuberculosis drug, and its metabolites induce of Kinic of histones through promoting the biosynthesis of isonicotinyl-CoA (Inic-CoA) in cells and mice (Fig. 2). Similar to the other acylations, Kinic is a dynamic regulatory process and could promote gene transcription by loosening the chromatin structure in the genome. Afterword, Kinic was reported to occur on non-histone proteins identified by global isonicotinylome analysis [82]. Notably, non-histone proteins Kinic is involved in multiple function pathways. The isonicotinyl-transferases, p300 and CBP function as the writers of histone Kinic, while histone deacetylase HDAC3 functions as a deisonicotinylase. [81]. Additionally, CBP and Tip60, have been reported to catalyze non-histone Kinic, and deisonicotinylases, including HDAC8 and HDAC6 could serve as deisonicotinylases in regulating non-histone proteins Kinic [82] (Table 1). So far, exploration of Kinic has been limited, but it is clear that Kinic, like other acylations, alters chromatin structure and affects gene expression. Moreover, it plays an essential role in regulating various cellular functions, such as cancer progression [82]. Nevertheless, further in-depth research is required to uncover its mysterious veil in regulating pathophysiology.

Collectively, the expanding repertoire of novel protein acylations, ranging from succinylation to isonicotinylation, exemplifies a sophisticated metabolic-epigenetic interface in which acyl-CoAs act as pivotal substrates for writers, erasers, and readers. These modifications dynamically regulate gene expression and protein structure and function, serving regulatory roles in biological processes such as metabolic flux and cellular responses. Consequently, it is imperative to further elucidate their molecular biological regulatory significance.

## Molecular biological significance of novel protein acylations

**Fig. 3:**
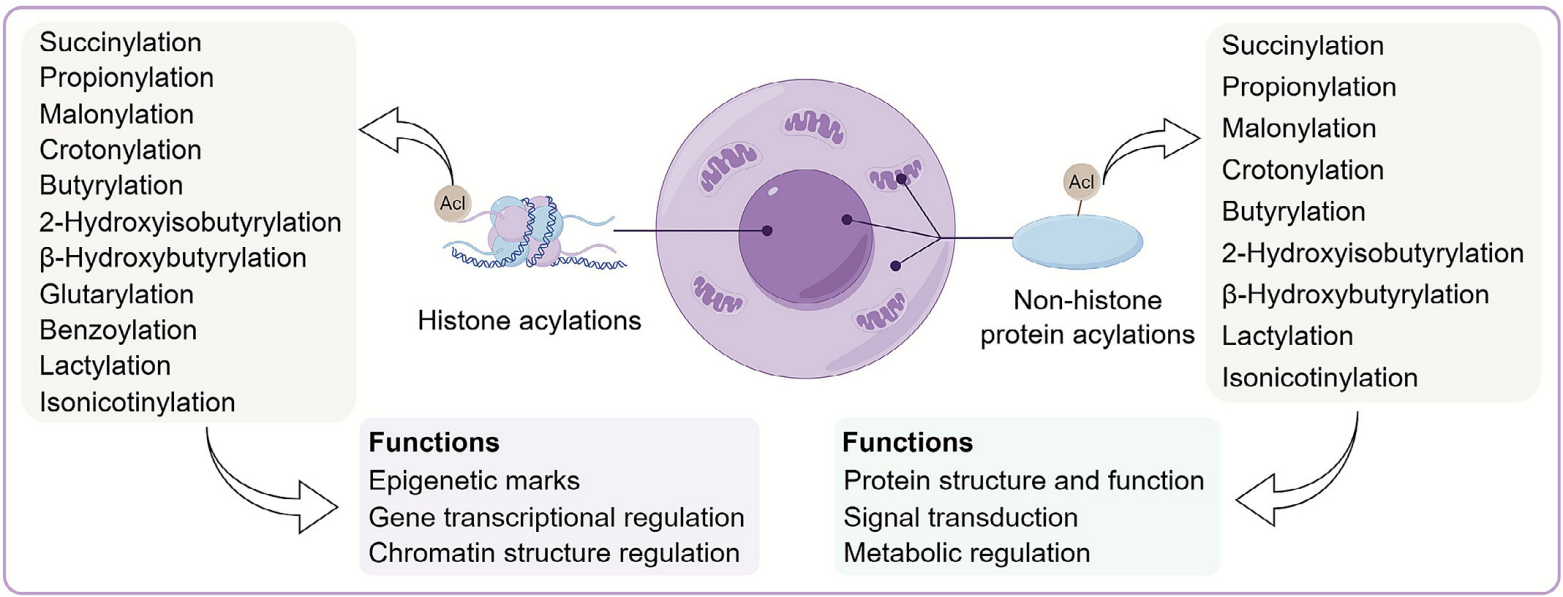
Biological functions of novel hitone and non-histione protein acylations. Novel histone acylations mainly affect gene transcription by regulating chromatin accessibility, serving as epigenetic marks. Novel non-histone protein acylations primarily alter protein structure and function, involving signal transduction and metabolic regulation. This figure is generated with FigDraw (https://www.figdraw.com).

**Fig. 4:**
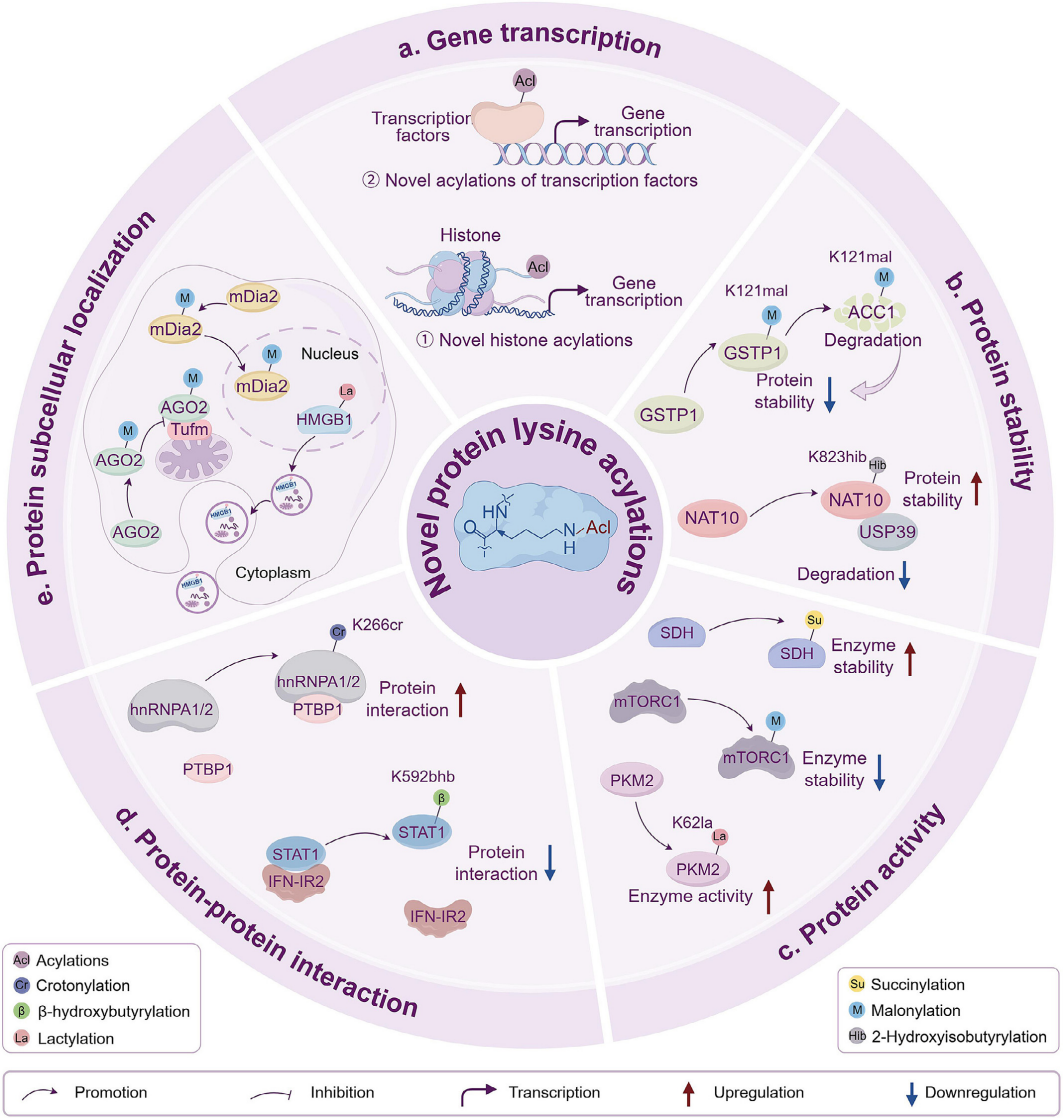
Molecular regulatory effects of novel protein acylations. Novel histone acylations and acylations of transcription factors precisely regulate gene transcription. Novel protein acylations influence protein characteristics and functions such as protein stability, enzyme activity, protein–protein interaction, and cellular translocation. (Abbreviation: GSTP: glutathione S-transferase P1; NAT10N-acetyltransferase 10; USP39: ubiquitin specific peptidase 39; SDH: succinate dehydrogenase; PKM2: pyruvate kinase M2; STAT1: signal transducer and activator of transcription 1; IFN-IR2: type-I interferon (IFN-I) receptor 2; PTBP1: polypyrimidine tract binding protein 1; hnRNPA1/2: heterogeneous nuclear ribonucleoprotein A1 and A2; AGO2: argonaute2; Tufm: Tu translation elongation factor, mitochondrial; mDia2: mammalian diaphanous 2). This figure is generated with FigDraw (https://www.figdraw.com).

Acylations predominantly regulate gene transcription, protein stability, enzyme activity, protein-protein interactions, and protein translocation. These molecular biological functions of novel acylations provide a fundamental molecular biology framework crucial for orchestrating epigenetic regulation, signal transduction, and metabolic adaption either in physiology and pathology (Fig. 3 and Fig. 4).

### Gene transcription

Recent advances have unveiled novel histone acylations as dynamic chromatin marks that directly modulate nucleosome architecture and transcriptional accessibility. Concurrently, novel non-histone acylations on transcription factors redefine their DNA-binding specificity and transactivation potential [83] (Fig. 4).

Histone acylation neutralizes the positive charge of specific lysine residues at the tail of histones, weakens the electrostatic interaction between histones and DNA, relaxes the nucleosome structure, loosens chromatin, increases chromatin accessibility, and provides more opportunities for transcription factors, RNA polymerases, and other proteins to bind to DNA, thereby promoting gene transcription [83]. Diverse novel histone acylations exhibit gene-activating roles. Histone succinylation, such as H3K23su, and H3K79su, destabilizes nucleosomes and reduces the affinity between DNA and histones, thereby promoting chromatin remodeling and gene transcription [84]. In addition, histone H3 propionylation at lysine 23 (H3K23pr) facilitates the transcription of a set of active developmental genes in leukemia mouse model [22]. Similarly, crotonylation of histone H3, especially H3K18cr, primarily marks active promoter regions, serving as a distinctive epigenetic indicator of crucial transcriptional regulators for gene transcription [42]. Histone butyrylation is also a key source of regulation on chromatin through impacting gene expression [44]. β-hydroxybutyrylation of histones is identified to be associated with the transcription of starvation-responsive genes [85]. Other novel histone acylations including glutarylation, lactylation, benzoylation, and isonicotinylation similarly alter nucleosome stability and correlate with active gene expression, collectively establishing acylations as versatile regulators of chromatin dynamics and transcriptional programs [67,81].

Emerging acylations are also redefining the regulatory landscape of transcription factors. These chemically diverse modifications directly modulate stability or activity of transcription factors, and DNA-binding affinity, thereby orchestrating context-specific gene expression programs in response to metabolic and environmental cues. For instance, β-hydroxybutyrylation of p53 inhibits the expression of downstream gene p21 and PUMA via attenuating p53 activity [59]. Together, these layered regulatory mechanisms establish a sophisticated metabolic-sensing framework for precise gene expression control.

### Protein stability

Emerging evidence highlights novel protein acylations dynamically modulate protein stability by altering structural integrity and susceptibility to degradation pathways, offering new mechanistic insights into the involvement of novel acylations in various cellular biological processes (Fig. 4). Intriguingly, certain acylations enhance structural rigidity and proteasomal resistance, others promote destabilization via charge alteration and degradation pathway activation, even homoacylation exhibits opposite effects on protein stability. Lys1523 malonylation of acetyl-CoA carboxylase 1 (ACC1) increases its enzyme activity and stability through inhibiting its ubiquitination degradation in ketogenic diet (KD)-improved non-alcoholic fatty liver disease (NAFLD) [86]. Conversely, malonylation of glutathione S-transferase P (GSTP1) diminished its stability through enhancing its degradation in SIRT5-knockout diabetic cardiomyopathy (DCM) mice [8]. Lysine butyrylation also plays a critical role in protein stability of HSP90, especially at Lys754, in chemoresistant esophageal squamous cell carcinoma (ESCC) cells [45]. Similarly, lysine 2-hydroxyisobutyrylation of N-acetyltransferase 10 (NAT10) in ESCC cells enhances its interaction with deubiquitinase ubiquitin specific peptidase 39 (USP39), resulting in increased NAT10 protein stability [51]. Lactylation of Apolipoprotein C2 (APOC2) at K70 stabilize it in non-small-cell lung cancer (NSCLC), promoting free fatty acids (FFAs) release, extracellular lipolysis and tumor metastasis [87]. These studies demonstrate that novel acylations represent a critical molecular mechanism for modulating enzyme biology, enhancing the understanding of the precise modulation of novel acylations on protein function.

### Enzyme activity

Novel lysine acylationsserve as dynamic regulators of enzymatic activity through bidirectional mechanisms, particularly for metabolism-related enzymes. While certain modifications (e.g., succinylation, lactylation) can enhance catalytic efficiency by modulating active site conformation, others (e.g., succinylation, malonylation) may suppress activity by disrupting substrate binding or oligomerization (Fig. 4). For example, in aging microglia, succinylation of succinate dehydrogenase (SDH) increases its enzymic activity to further promote TCA cycle and succinyl-CoA the production, while trifunctional enzyme subunit alpha (ECHA) succinylation reduces its activity and leads to β-oxidation inhibition-induced lipid accumulation [9]. In addition, malonylation of mTOR at Lys1218 impairs mTOR complex 1 (mTORC1) kinase activity, contributing to angiogenic defects in fatty acid synthase (FASN)-knockdown endothelial cells (ECs) [30]. Moreover, lactylation influence enzymatic activity, especially for metabolic enzymes such as pyruvate kinase M2 (PKM2) [88,89]. Additionally, NSUN2 activity is enhanced by lactate-mediated lactylation at lysine 508, which then targets glutamate-cysteine ligase catalytic subunit (GCLC) mRNA to facilitates GCLC m^5^C formation and mRNA stabilization [79]. These findings position novel protein acylations as versatile post-translational switch that directly couples cellular metabolism with enzymatic regulation via modulating enzyme activity.

### Protein-protein interaction

Novel lysine acylations dynamically rewire protein-protein interaction networks by modulating interfacial charge, hydrophobicity, and structural conformation [90]. While modifications like crotonylation and 2‐hydroxyisobutyrylation can enhance complex assembly through allosteric stabilization. For instance, crotonylation of polypyrimidine tract binding protein 1 (PTBP1) at K266 enhances the interaction of PTBP1 with heterogeneous nuclear ribonucleoprotein A1 and A2 (hnRNPA1/2) to form complex, thus affecting the *PKM* alternative splicing in colorectal cancer (CRC) cells [10]. Similarly, NAT10 Khib modification promotes its interaction with deubiquitinase USP39, resulting in decreased NAT10 degradation and increased protein stability in ESCC [51]. β-hydroxybutyrylation of sorting nexin-9 (SNX9), a key regulator of MDV biogenesis, increases SNX9 interaction with inner mitochondrial membrane (IMM)/matrix proteins and eventually promoting the formation of IMM/matrix Mitochondrial-derived vesicles (MDVs) [91]. While others modifications such as succinylation and β-hydroxybutyrylation disrupts binding interfaces via steric hindrance or electrostatic repulsion. Succinylation of valosin-containing protein (VCP) at K658 inhibits its interaction with mitofusin 1 (MFN1) and thus inhibits TNF-α-induced mitophagy of bone marrow mesenchymal stem cells (BMMSC) [92]. This regulatory plasticity on protein-protein interaction allows novel acylations to serve as metabolic sensors that spatially orchestrate cellular machinery.

### Protein subcellular localization

The subcellular distribution and trafficking of proteins are dynamically governed by novel acylations, which modulate compartment-specific targeting through diverse structural and charge-based mechanisms. These modifications direct proteins to critical organelles including nuclear, cytoplasm, mitochondria, membranes, and beyond while concurrently regulating interorganelle transfer (Fig. 4). For instance, malonylation of mDia2 drives actin assembly in HCC cells and promotes its nuclear translocation to facilitate metastasis [93]. Lactylation also can promotes protein nuclear localization through enhancing protein binding affinity [94]. Conversely, malonylation of NCL at Lys124 and Lys398 directs its translocation from the nucleolus to nucleoplasm and cytoplasm for binding mRNA and facilitating protein translation in HCC [95]. HMBG1 lactylation in macrophage also induces its translocation from nucleus to the cytoplasm and releases into the extracellular space via exosome [96]. Notably, argonaute2 (AGO2) malonylation inhibits the functional subcellular properties of AGO2 and reduces its importing into mitochondria in diabetic cardiomyopathy [29]. Similarly, lactylation of Tu translation elongation factor, mitochondrial (Tufm) at K286 impedes its interaction with Tomm40 on mitochondria and the mitochondrial localization, leading to mitophagy in traumatic brain injury [11]. These studies elucidate the diverse roles of novel acylations in altering the subcellular localization of proteins. Such spatial control enables novel acylations to orchestrate metabolic and signaling adaptations at the cellular level.

Altogether, novel protein acylations constitute a versatile regulatory layer that dynamically orchestrates fundamental cellular processes including gene transcription, protein stability, enzyme activity, protein interactions, and subcellular trafficking through precise chemical modifications of lysine residues. These modifications act as molecular sensors that translate metabolic fluctuations into functional adaptations, thereby integrating cellular metabolism with epigenetic reprogramming, signaling cascades, and organelle dynamics. Their bidirectional and context-specific impacts underscore a sophisticated mechanism for maintaining cellular homeostasis in physiology and pathology.

## Involvement of novel protein acylations in AD

**Fig. 5:**
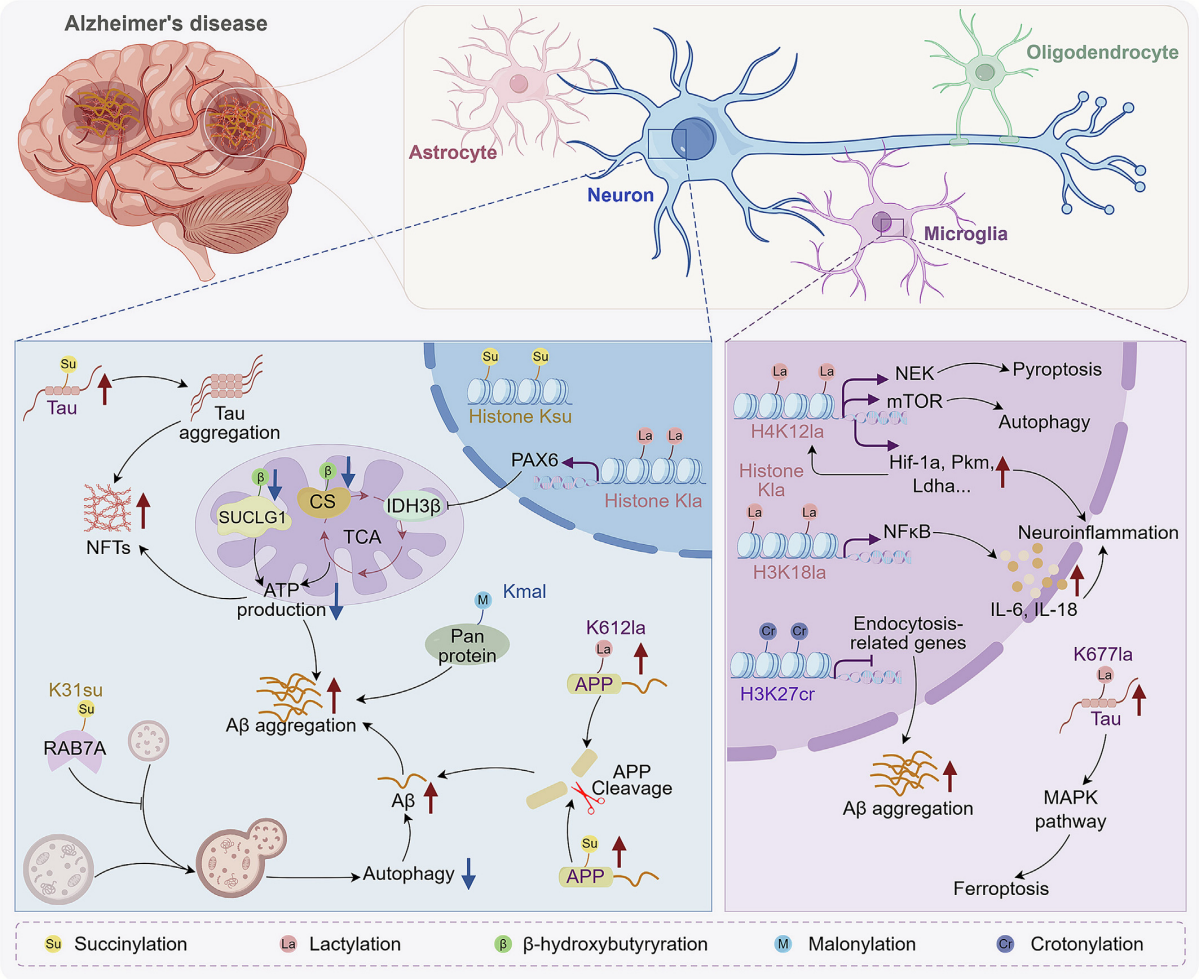
Pathological regulatory mechanisms of novel protein acylations in AD. Novel protein acylations, especially succinylation, crotonylation, malonylation, β-hydroxybutylation, and lactylation, contribute to AD pathology via regulating various cellular biological processes including neuroinflammation, metabolic dysfunction, and programmed cell death of neurons and microglia. (Abbreviation: NFTs: neurofibrillary tangles; RAB7A: Ras-associated protein 7a; IDH3β: isocitrate dehydrogenase 3β; APP: amyloid precursor protein; NEK7: never in mitosis gene a related kinase 7; mTOR: mechanistic target of rapamycin; Hif-1α: hypoxia-inducible factor 1-alpha; Pkm: pyruvate kinase M; Ldha: lactate dehydrogenase a; NFκB: nuclear factor kappa-light-chain-enhancer of activated B). This figure is generated with FigDraw (https://www.figdraw.com).

**Fig. 6:**
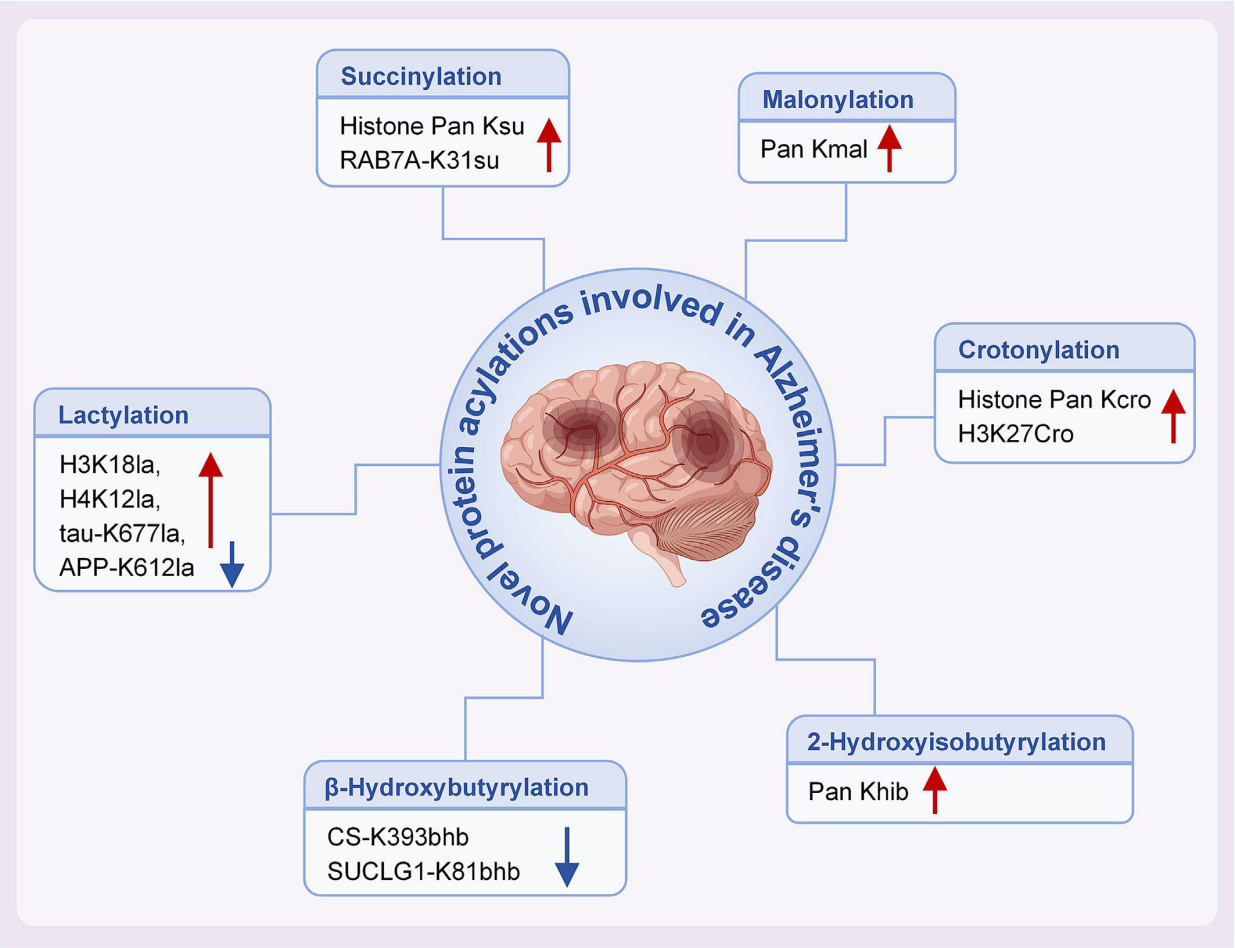
Involvement of altered novel protein acylations in AD. Various dysregulated novel protein acylations are involved in AD, such as upregulated lactylation, succinylation, crotonylation, malonylation, and 2-hydroxyisobutyrylation; While downregulated β-hydroxybutyrylation exists in AD pathology. (Abbreviation: RAB7A: Ras-associated protein 7a; CS: citrate synthase; SUCLG1: succinate-CoA ligase subunit alpha).

In recent years, the relationship between acylation modifications and AD has garnered increasing scholarly attention, with significant progress being made, particularly in light of the growing body of research on novel post-translational acylation modifications (Fig. 5 and Fig. 6, and Table 2). The brain, as one of the most metabolically active organs, relies heavily on a continuous supply of energy substrates due to its limited energy reserves. Disruptions in glucose metabolism and other metabolic pathways, whether affecting the entire brain or specific cell types, are closely associated with AD. In this context, the significance of metabolism-derived post-translational acylations in brain function is becoming increasingly evident. Understanding the role of novel post-translational acylations in AD could yield valuable insights into the metabolic underpinnings of the disease, aid in the identification of therapeutic targets, and potentially inform future treatment strategies.

**Table 2:** Novel protein acylations identified in AD.

Novel acylation modifications	Modified proteins and sites	Models	Biological functions or mechanisms	Refs
Succinylation	RAB7A-K31su	Cadmium -exposed FAD^4T^ mice; Cadmium-induced N2a cells	Inhibits RAB7A activity, which contributes to autophagic flux blockade	[18]
	APP, Tau-Pan Ksu	Brain tissue of AD patients; sTg19959 mice	Disrupts normal proteolytic processing of APP and promotes Aβ accumulation as well as enhances tau aggregation to tangles and impaired microtubule assembly	[97]
	Tau-K311su	N/A	Influences both the physiological and pathological interactions of tau with microtubule	[98]
Malonylation	Pan Kmal	N2a/APP_swe695_ cells	N/A	[99]
Crotonylation	H3K27cr	APPswe/PS1dE9 double transgenic mice; U251 cells	Facilitates expression of multiple endocytosis-related genes and mediates Aβ clearance	[34]
	Pan Kcr	N2a/APP_swe695_ cells	N/A	[99]
β-hydroxybutyrylation	CS-K393bhb, SUCLG1-K81bhb	APP/PS1 mice; HT22 cells	Kbhb modifications and enzymatic activities decreases, which associated with increased Aβ plaque pathologies and microgliosis	[58]
Lactylation	H4K12la	Brain tissues of AD patients; 5xFAD mice; Primary microglia and BV-2 cells	Positive feedback promotes glycolysis, exacerbates microglial dysfunction and Aβ pathology	[109]
	H4K12la	APP/PS1 mice; BV-2 cells	Elevates the expression of NEK7, triggering pyroptosis of microglia	[101]
	H4K12la	APP/PS1 mice; BV-2 cells	Impairs the autophagic flux in microglia by activating the mTOR signal	[102]
	H3K18la; H4K12/18la	Human post-mortem cortical tissue sections; 5xFAD mice; N2a cells	Enhances the expression of PAX6, which in turn inhibits the transcription of IDH3β, forming positive feedback inhibition loop of IDH3β-lactate-PAX6-IDH3β and accelerating progression of AD	[106]
	H3K18la	APP/PS1 and FAD^4T^ AD modeling mice; BV-2 cells	Stimulates the NF-κB signaling pathway and upregulates SASP components IL-6 and IL-8, exacerbating neuroinflammation	[103]
	H3K18la	D-gal/AlCl3-induced AD-like mice; BV-2 cells	Improves cognitive dysfunction and neuroinflammation in mice	[104]
	APP-K612la	APP23/PS45 double-transgenic AD mice; APP_swe695_ cells	Regulates transcription associated with APP trafficking and metabolism, promotes the APP endosomal-lysosomal degradation process associated with CD2AP, inhibiting Aβ generation and slowing down cognitive deficits	[107]
	Tau-K677la	APP/PS1 mice; BV-2 cells	Promotes ferroptosis by regulating iron metabolism factors like NCOA4 and FTH1, deteriorating memory and learning skills	[108]

N/A indicates not available. RAB7A: Ras-associated protein 7a; APP: amyloid precursor protein; NEK7: never in mitosis gene a related kinase 7; PAX6: paired box 6; IDH3β: isocitrate dehydrogenase 3 beta; SASP: senescence-associated secretory phenotype; NCOA4: nuclear receptor coactivator 4; FTH1: ferritin heavy chain 1.

### Succinylation in AD

Succinylation has been identified to be intricately linked with the pathogenesis of AD (Fig. 5 and Fig. 6, and Table 2). In cadmium-exposed FAD^4T^ mice, the hampered expression of SIRT5 induces the succinylation of Ras-associated protein 7a (RAB7A) at lysine 31 (K31), which inhibits RAB7A activity and contributes to autophagic flux blockade, eventually causing Alzheimer’s disease-like pathology including amyloid-β (Aβ) deposition and memory deficits [18]. Moreover, altered succinylation has been identified on mitochondrial proteins, APP and tau in succinylomes and proteomes from brains of individuals with AD. The soluble and insoluble APP derivatives and tau in AD mice exhibit high levels of succinylation, which disrupts normal proteolytic processing of APP and promotes Aβ accumulation as well as enhances tau aggregation to tangles and impaired microtubule assembly [97]. Succinylation of tau K311 perturbs tau microtubule binding, may impact both the physiological and pathological interactions [98]. These evidences suggest the significance of succinylation in AD pathology, implying its potential as biomarker and therapeutic target.

### Crotonylation in AD

Crotonylation has been identified to be involved in the pathogenesis of AD, but the research is limited (Fig. 5 and Fig. 6, and Table 2). The repressed expression of nuclear paraspeckle assembly transcript 1 (NEAT1) in the early stage of AD up-regulates H3K27 crotonylation (H3K27Cro), which locates nearby to the transcription start site of endocytosis related genes, thereby inhibiting Aβ clearance [34]. Additionally, high crotonylation level has also identified in Aβ-induced neurotoxicity in N2a/APP695swe cells [99]. Overall, these findings indicate that crotonylation is a promising therapeutic target for AD. However, the exact regulatory role of crotonylation in the pathogenesis of AD is still a lack of clarity, which needs to be further investigated and interpretated in the future study.

### β-Hydroxybutyrylation in AD

β-Hydroxybutylation serves as potential regulator of AD pathology (Fig. 5 and Fig. 6, and Table 2). A study on 8-month-old and 2-month-old APP/PS1 AD model mice using 4D label-free β-hydroxybutylation quantitative proteomics found decreased Kbhb modification levels, particularly in TCA cycle enzymes and SUCLG1 [58]. Notably, the Kbhb modifications and enzymatic activities of CS and SUCLG1 were dramatically reduced, diminishing ATP production. A ketogenic diet, enhanced Kbhb modifications and enzymatic activities of CS and SUCLG1, and increased ATP production, thereby reducing Aβ pathologies and microgliosis in APP/PS1 mice. This study suggests the importance of protein Kbhb modification in maintaining energy metabolism and even offering intervention strategies for AD treatment.

### Lactylation in AD

Increasing evidence has revealed the involvement of hitone lactylation in the pathogenesis and improvement of AD (Fig. 5 and Fig. 6, and Table 2). In the brains of AD patients and AD transgenic mice, glucose metabolism is impaired, including glycolysis, oxidative phosphorylation, and TCA cycle. The elevated glycolysis in microglia of brain of 5xFAD model mice and AD patients triggers histone lactylation, particularly H4K12la, in turn facilitates transcriptional activation of glycolytic genes including PKM2, LDHA and hypoxia-inducible factor-1α (HIF-1α), establishing a positive feedback loop of glycolysis/H4K12la/PKM2 and intensifying microglial dysfunction. However, knocking down PKM2 to block the process ameliorates Aβ pathology and cognitive and memory deficiency in AD mice [100]. Furthermore, H4K12la in microglia promotes the transcription of never in mitosis A-related kinase 7 (NEK7), an inducer of activation of NLRP3 inflammasome, initiating neuroinflammation and exacerbating impairment of learning and memory in AD mice [101]. Similarly, cigarette smoke-induced lactate overproduction promotes the transcriptional activation of NLRP3 and subsequently impairs mTOR signally-mediated autophagic flux in microglia, leading to the increased plaque deposition and microglial activation [102]. Consistently, the significantly elevated lactate in senescent microglia in aged mice (FAD^4T^) and APP/PS1 mice leads to histone Kla, particularly H3K18la, enhancing the expression of inflammatory factors such as IL-6 and IL-8, as well as the activation of NFκB signaling pathway, eventually exacerbating neuroinflammation [103]. Conversely, exercise-induced H3K18la in microglia improves neuroinflammation and cognitive impairment in AD mice [104]. Intriguingly, *Porphyromonas gingivalis* msRNA P.G_45033 causes the production of Aβ in macrophages of periodontal tissue by promoting glycolysis and histone lactylation, enhancing the risk of AD occurrence [105]. In addition, the downregulated isocitrate dehydrogenase 3β (IDH3β), a key enzyme of TCA cycle, in neurons leads to histone lactylation, especially H3K18la and H4K12/18la, and the subsequent transcriptional activation of paired-box gene 6 (PAX6), a repressive transcription factor for IDH3β, forming IDH3β-lactate-PAX6-IDH3β feedback loop and exacerbating the progression of AD [106].

Besides, non-histone lactylation is identified as an essential contributor of AD pathology. For instance, in APP23/PS45 double-transgenic mouse model of AD, lactylation of APP at lysine 612 (K612) facilitates its trafficking and metabolism by inhibiting its binding to BACE1and the subsequent cleavage, accompanied with endosomal-lysosomal degradation, thereby suppressing Aβ generation and improving spatial learning and memory impairment [107]. Moreover, lactylation of tau at lysine 677 (K677la) orchestrates AD by modulating ferritinophagy and ferroptosis. Inhibition of tau K677la in brain tissues of the AD mice and Aβ-induced BV2 cells by tau mutation regulates iron metabolism factors like NCOA4 and FTH1, and subsequently impedes ferroptosis [108]. These findings emphasize the link between glucose metabolism disorders and lactylation, as well as the key role of lactylation in the occurrence and development of neuroinflammation and AD, implying the possibility of targeting glucose metabolism and lactylation as therapeutic strategies for AD and senescence.

Moreover, the decreased levels of malonylation, and 2-hydroxyisobutyrylation have also been identified to be related with the damage of in AD neurons, and may be restored by treatment of crocin [99].

Taken together, various novel post-translational acylation modifications have been proven to play a significant role in AD pathology and even in the amelioration of AD, suggesting that they may serve as key biomarkers and potential intervention targets for the diagnosis and treatment of AD.

## Diagnostic and therapeutic potential of novel acylations

Novel acylation orchestrates diverse cellular processes through multiple molecular regulation, especially gene expression, metabolic reprogramming, epigenetic remodeling, and signaling transduction. The dynamic nature and metabolic sensitivity of novel acylation modifications position them as compelling candidates for diagnostic biomarkers and therapeutic targets in various diseases such as AD. Comprehensive investigation into novel acylation-mediated molecular networks uncovers novel diagnostic biomarkers and therapeutic targets for disease, positioning this regulatory mechanism as a pivotal frontier for clinical application and targeted disease management strategies. Critically, the enzymatic regulation including targeting writers, and erasers of these modifications provides tangible molecular nodes for intervention.

### Biomarkers for disease diagnosis involving novel acylations

The potential of novel acylations as diagnostic biomarkers stems from their pathology-specific dysregulation and potential detectability in accessible biofluids. AD brain tissues and models exhibit distinct acylation profiles. For instance, decreased Kbhb on TCA cycle enzymes (CS-K393bhb, SUCLG1-K81bhb) correlates with impaired ATP production and Aβ pathology in APP/PS1 mice [58]. Specific histone lactylation marks (e.g., elevated H4K12la in microglia) are tightly coupled to pro-inflammatory glycolytic shifts and disease progression in human AD patients and 5xFAD mice [101]. Pan-succinylation increases on APP and tau in AD brains disrupt proteostasis and promote aggregation [97]. These specific modifications represent molecular fingerprints of ongoing pathological processes.

While direct brain measurement is ideal, the stability of certain acylated peptides/proteins raises the possibility of detecting them in cerebrospinal fluid (CSF) or potentially blood-based exosomes. For example, Lactylation of HMGB1 promotes its extracellular release via exosomes [96]. Similar mechanisms could export other acylated neuronal or glial proteins into the CSF. Technological advances in mass spectrometry-based proteomics (e.g., pan-antibody enrichment coupled with high-resolution LC-MS/MS) and potentially the development of modification-specific antibodies could enable the quantification of these signatures as minimally invasive diagnostic or prognostic tools [110]. Furthermore, by employing machine learning techniques on databases, the expression levels of novel acylations-related genes have been identified to construct a gene signature, which may serve as a biomarker for clinical prognosis and the responsiveness to clinical treatments.

As novel acylations such as succinylation, lactylation, and β-hydroxybutyrylation, directly reflect the levels of their precursor metabolites succinyl-CoA, lactate, and β-hydroxybutyrate, respectively, monitoring these modifications offers an integrated readout of functional brain metabolic state, which is profoundly disturbed in AD. This provides more direct insight into metabolic health than measuring metabolite levels alone, as it captures the downstream functional consequence on protein networks.

### Targeted therapeutics involving novel acylations

Targeting the regulatory machinery of novel acylations presents promising therapeutic avenues for AD, focusing on restoring metabolic-epigenetic-protein homeostasis. Modulating regulatory enzymes hold significant potential in intervening in diseases. For instance, reactivation of SIRT5 via small-molecule activators) could mitigate pathological hyper-succinylation related autophagy in AD models [18]. Selectively inhibiting hyperactive acyltransferases could also be beneficial. For example, targeting inhibition of p300/CBP, a major writer for multiple detrimental acylations, might alleviate H4K12la-mediated microglial dysfunction [109].

Modulating the availability of acyl-CoA donors offers an indirect but powerful strategy. Elevating BHB levels via ketogenic diets or BHB esters increases Kbhb on metabolic enzymes (CS, SUCLG1), restoring their activity, boosting ATP production, and reducing Aβ pathology and microgliosis in APP/PS1 mice [58]. This provides direct proof of concept for metabolic intervention via acylation modulations. Strategies to reduce lactate production or enhance its clearance via inhibiting glycolysis or monocarboxylate transporters (MCTs) inhibitors might dampen pathogenic lactylation driving neuroinflammation [104]. Similarly, modulating succinyl-CoA flux via TCA cycle modulators could impact pathogenic succinylation.

Moreover, identifying specific reader proteins for pathogenic acyl marks, such as ENL for H3K9bhb, or DPF for lactylation, could lead to develop inhibitors that disrupt the transmission of the acylation signal to transcriptional or functional outcomes, such as aberrant microglial activation or impaired neuronal metabolism [61,111].

## Conclusion and perspectives

This comprehensive review synthesizes the rapidly evolving field of novel protein acylation modifications and their profound implications in AD pathogenesis. We have detailed the enzymatic machinery including donor and acyl source, regulatory enzymes including writers, erasers, readers, the diverse molecular mechanisms such as transcriptional regulation, protein stability, enzyme activity, protein interactions, subcellular localization. Moreover, we have summarized the specific roles of key acylations such as succinylation, crotonylation, β-hydroxybutyrylation, and particularly lactylation in driving core AD pathologies including Aβ accumulation, tau hyperphosphorylation, neuroinflammation, metabolic dysfunction, and programmed death of neurons and glial cells. Collectively, these findings establish novel acylations as crucial metabolic-epigenetic regulators that intricately link cellular metabolic states, particularly those involving mitochondrial function and glycolysis, to gene expression and protein function derangements central to AD. This integrated perspective significantly advances our understanding of AD beyond the classical amyloid and tau hypotheses, highlighting a dynamic layer of post-translational control.

The significance of this synthesis lies in its identification of novel acylations as pivotal nodes connecting well-established AD risk factors like metabolic dysregulation and aging to downstream neurodegenerative processes [112]. By elucidating how novel acylations directly modify protein function and chromatin states, this review fills a critical knowledge gap regarding the molecular mechanisms bridging metabolism, protein PTMs and neurodegeneration. This has substantial implications for both basic research and clinical translation, providing a mechanistic rationale for exploring metabolic interventions (e.g., ketogenic diets), identifying novel druggable targets within the acylation regulatory network such as specific acyltransferases/deacylases, and offering potential avenues for developing diagnostic biomarkers based on specific acylation signatures [113,114].

A critical mechanistic insight underlying the diverse functional consequences of these acylations lies in their ability to fundamentally alter the physical and chemical properties of the modified lysine residues. Each acyl group confers a distinct set of properties, such as charge, hydrophobicity, and steric bulk, which directly influence protein conformation and function. For instance, succinylation introduces a large, negatively charged moiety that can neutralize the positive charge of a lysine, potentially disrupting electrostatic interactions in histone-DNA binding or protein-protein interfaces [115]. In contrast, crotonylation, while also removing the positive charge, adds a hydrophobic and planar conjugated system that may facilitate unique hydrophobic interactions or act as a “reader” module [116,117]. β-Hydroxybutyrylation and lactylation, bearing a hydroxyl group, introduce both hydrophobicity and potential for hydrogen bonding, creating a unique chemical environment that could modulate protein solubility and recruitment of specific effector proteins [[118], [119], [120]]. These alterations in charge, hydrophobicity, and acidity/basicity directly govern downstream effects, including changes in protein stability, enzymatic activity, and the formation of biomolecular condensates, thereby providing a physicochemical basis for the distinct pathological roles of each acylation type in AD.

While this review encompasses the major currently characterized novel acylations and their established roles in AD models, it is necessarily limited by the exclusion of some emerging or less-studied modifications due to space constraints and the nascent nature of the field. Furthermore, the depth of discussion on cell-type-specific effects (e.g., neuron vs. microglia vs. astrocyte) and regional heterogeneity within the brain could be expanded as more data becomes available [121]. The reliance primarily on preclinical models including cell lines and transgenic mice also necessitate caution in extrapolating findings directly to human AD, highlighting a key limitation. These gaps mean that the full spectrum of acylations involved in AD and their precise spatiotemporal dynamics in the human brain remain incompletely mapped.

Future research should prioritize several key directions: (1) Employing advanced spatial multi-omics and cell-type-specific profiling in human post-mortem brain tissue across different AD stages to define the precise landscape of dysregulated acylations [122]; (2) Elucidating the complex crosstalk between different acylation types and with other PTMs, such as ubiquitination, glycosylation, on the same AD-related protein substrates including tau, and APP [123]; (3) Developing highly specific modulators including activators or inhibitors for key regulatory enzymes including writers, readers, and erasers, and rigorously testing their therapeutic efficacy and safety in diverse AD models [124]; (4) Establishing robust, sensitive, and specific methods to detect and quantify pathologically relevant acylation marks in CSF, plasma, or exosomes for early diagnosis and disease monitoring [125]; (5) Investigating the impact of gut microbiome-derived metabolites on brain protein acylation and AD progression [126]. Pursuing these avenues will be essential to translate the mechanistic insights reviewed here into tangible clinical benefits.

In conclusion, this review underscores the fundamental role of novel protein acylation modifications as critical integrators of metabolic state and cellular function in the brain, positioning them as central players in AD etiology and progression. By systematically defining their mechanisms, molecular impacts, and pathological significance, this work provides a vital foundation for future exploration, offering exciting new perspectives for developing innovative diagnostic tools and targeted therapeutic strategies to combat this devastating neurodegenerative disorder. The burgeoning field of novel protein acylations holds promise for revolutionizing our understanding and treatment of AD.

## Compliance with ethics requirements

Our article does not contain any studies with human or animal subjects.

## CRediT author contribution statement

Yue Yang, Mingyan Liu and Minjie Wei designed the manuscript. Yue Yang and Zhihua Yu wrote the manuscript. Ziyi Zhang, Yuehua Zhang and Rui Gao did literature search. Yue Yang, Xin Zhong and Ke Du drafted figures. Linchi Jiao and Yuqiang Wu listed the tables. All the co-authors revised the manuscript. All authors listed have made a substantial contribution to this study and approved the article.

## Declaration of competing interest

The authors declare that they have no known competing financial interests or personal relationships that could have appeared to influence the work reported in this paper.
